# A Pilot Study of the Short Term Effectiveness and Safety of Amniotic Fluid in Severe Dry Eye Disease

**Published:** 2019

**Authors:** Ming CHEN, Chao Kai CHANG, Szu Yuan LIN, Mindy CHEN

**Affiliations:** 1 John A. Burns School of Medicine, University of Hawaii, USA

**Keywords:** Amniotic Fluid, Severe Dry Eye Disease, Soft Contact Lens, Dry Eye Workshop

## Abstract

The aim of this study was to evaluate if amniotic fluid (AF) mixed with artificial tears or soaked with a soft contact lens (SCL) as a treatment for severe dry eye disease (DED) would improve its signs or symptoms. In this retrospective pilot study 22 consecutive eyes of 11 patients with level 3 DED classified by DEWS 1 (Dry Eye WorkShop 1 2007), were included in the study between June 1 and September 30 in 2017. The study was conducted before DEWS II (Dry Eye WorkShop II 2017), which was published in October 2017. Therefore, DEWS II was not adopted for this study. Soft Contact Lens Acuvue Oasys of Plano with 8.8 base curve and 14 mm diameters by Johnson and Johnson were used to soak in FloGraft, which is an AF, for 30 minutes before placing in 12 eyes in Group 1. The contact lenses were placed in the left eye for 1 week. In Group 2, 10 eyes used 6 mL of artificial tears mixed with 0.25 mL of AF, which were applied to the eye four times a day for 1 week. No other eye medications were used. The eyes that were included had diffuse punctate staining and fast tear breaking times of <5 seconds with severe ocular symptoms (DEWS 1 level 3-4). Photos of fluorescein stain corneas before the treatment and 1 week after the treatment were used to compare the distribution of punctate staining as the objective outcomes for signs. Several questions adopted from the Ocular Surface disease Index (OSDI) about subjective symptoms before and after the treatment were asked, and documented on the chart. Improvement either in symptoms or signs or both of DED after 1 week at follow-up examination was recorded. Group 1, with SCL 46% had improvement after 1 week and Group 2, with artificial tears 50% had improvement after 1 week. Improvement means either symptom relief or comparatively decreased distribution of punctate staining on the cornea or both. No cases had inflammation, infection, irritation or blurred vision. We concluded that FloGraft as an AF can safely improve the symptoms or the signs of severe DED either as a mix with artificial tears or soaked with SCL by about 50% in this case series without evidence of irritation, inflammation or blurred vision in the short term.

## INTRODUCTION

The incidence of dry eyes in the population has been reported to range from 7.4% to 33.7% with increased incidence in females and the elderly [1]. Level 3 dry eye disease (DED) defined by Dry Eye WorkShop (DEWS) [2] is diffuse punctate staining with erosion or filament on cornea with fast tear breaking time (TBUT) of up to < 5 seconds with severe symptoms [2]. The treatments for Level 3 DED recommended by DEWS 1 are autologous serum or umbilical cord serum, contact lenses or permanent punctal occlusion. These treatments recommended by DEWS 1 have some setbacks; the autologous serum or umbilical cord serum can be expensive to acquire, because production of serum eye drops requires much time and labor [3]. Contact lens can be difficult for some patients to wear and tolerate. Treatment with permanent punctum occlusion may cause epiphora [4].

New treatment option such as using amniotic membrane has been increasing in popularity. However, the amniotic membranes are expensive and uncomfortable for patients [5]. Vision can be affected for daily life as shown in Table 1.

**Table 1:** Comparison between Amniotic Membrane and Amniotic Fluid

	Amniotic membrane ( Prokera, Bio-tissue, Miami, Florida, USA) [5]	Amniotic fluid (FloGraft, (Applied Biologics Scottsdale, AZ USA) [6]
Cost	More expensive about $600	Less expensive about $200
Comfort	Less comfort/with big ring	More comfort/fluid
Vision	Translucent (blurred)/hazy Media	Transparent (clear)

Human amnion and amniotic fluid (AF) play a significant role in the development and protection of the body’s complex physiological system [6, 7]. AF contains native nutrients and growth factors including hormones such as Transforming Growth Factors Beta1 (TGFß1), Endothelial Growth Factors (EGF), and Fibroblast Growth Factors (FGF). The composition of AF changes as the gestation progresses. Hamid et al. reported isolation of pluripotent stem cells from full-term AF [8]. It has been demonstrated in peer-reviewed journals that the natural biologic properties of the placenta have been shown to promote tissue repair and management of localized inflammation [9-11]. While AF may be less expensive and comfortable to apply, it has the ability for a long term combined therapy with amniotic membranes or traditional therapy for DED. Therefore, AF may be a good alternative and is worthwhile to investigate further. Hence, the aim of this study was to assess whether AF either mixed with artificial tears or soaked with a soft contact lens (SCL) as a treatment for severe DED would improve its signs and or symptoms.

## METHODS

This is a retrospective pilot study of 22 eyes of 11 subjects in Dr. Chen’s clinic in Honolulu, Hawaii, the United States in 2017 (from January 2017 to December 2017). This study was approved by the Institutional Review Board (IRB) of the University of Hawaii. All patients signed written informed consents for the treatment. The study was performed in accordance with the Declaration of Helsinki and other applicable guidelines and regulations. Ethical approval has been received accordingly. Sterile SCL Acuvue Oasys of Plano with 8.8 base curve and 14 mm diameters by Johnson and Johnson were used to soak in FloGraft, which is an AF 30 minutes before placing in 12 eyes in group 1 and treated for one week. Soft contact lenses were soaked with 0.25 mL FloGraft for one hour. In Group 2, 10 eyes were treated with 6 mL of sterile artificial tears mixed with 0.25 mL of FloGraft four times a day for one week. All eyes had level 3-4 dry disease classified by DEWS 1 with diffuse Superficial Punctate Keratitis (SPK) and fast Tear Breaking up Time (TBUT) (<5 second) with severe symptoms (Level 3-4). Photos were taken with fluorescein stain on cornea before the treatment, and one week after it, to compare distribution of punctate staining. Questions were asked for the symptom of DED adopted from Ocular Surface Disease Index (OSDI) questionnaires before and after the treatment [12]. “FloGraft® is an AF regulated under Section 361 of the Public Health Status Act and meets the criteria of Title 21 Code for Federal Regulation (CFR) 1271.3 and 1271.10” Applied Biologics (Applied Biologics Scottsdale, AZ USA). Outcome measures of this study were improvement of symptoms or signs or both after one week at a follow-up examination. Patients subjectively had one or more less complaints of the following symptoms such as blurring of vision, pain, dryness, itching, red eye or mucus discharge, which were asked and recorded. These answers were considered as the symptom improvement. 

Ocular Surface Disease Index questionnaires scores were not completed because some elderly non-English speaking patients in this study had difficulties to complete the questionnaire. Less distribution of punctate staining and slower TBUT compared to that before the treatment on cornea were identified and documented by photo as the sign of improvement. Data collected and descriptive analysis used to present frequency and percentages of findings in each group.

## RESULTS

Overall 22 eyes of 11 Asian subjects with mean ± standard deviation (SD) age of 70±3.21 years including 2 males (18%) and 9 (82%) females were evaluated in this study. Status of dry eye signs and symptom before and after the treatment with AF (FloGraft) soaked SCL in Group 1 and AF (FloGraft) with artificial tears in Group 2 is shown in detail in Table 2. As shown in Table 2, in Group 1, diffuse corneal staining improved in 5 eyes (42%) and in Group 2 in 5 eyes (50%). In both Groups, TBUT, pain, red eye, dry feeling, blurring of vision, secondary tearing, itchiness, and mucus discharge improved in 50% of eyes which means in 6 eyes in Group1 and 5 eyes in Group2. Taken as a whole, in Group 1, 46% had improvement in signs or symptoms of DED after one week and in Group 2, 50% had improvement after one week. None of the cases had infection, reaction or inflammation needed to be treated with antibiotics or steroid.

## DISCUSSION

In the current study after one week of treatment, the AF with SCL had 46% improvement and the AF with artificial tears had 50% improvement. 

Dry eye disease is a complex and multifactorial eye disease. It is very difficult to treat in level 3-4 as various treatments have been provided without sufficient effectiveness. There is a recent review of amniotic membranes extracts (AME) and amniotic extract eye drops (AMEED) in treating severe superficial cornea eye disease by Murri [13]. The review showed benefits of AMEED in treating superficial cornea disease. The two complete clinical trials on AMEED demonstrated reduced cornea staining, reduced inflammation, reduced pain and acceleration of corneal epithelium healing. However, there was no study on AF. The two completed clinical trials on AMEED were reported on ClinicalTrials.gov. The first is “Utilization of Amniotic Membrane Extract Eye Drop (AMEED) on Human Corneal Healing” and the other is “The Improvement of Limbal Stem Cell Deficiency (LSCD) in Unilateral Stem Cell Damage by AMEED.” Both studies were from Labafi Nejad Eye Research Center in Tehran, Iran [14, 15]. Another recently published paper by Shayan et al. indicated that AMEED increases Limbal stem cell (LSC) proliferation and accelerates corneal epithelium healing without adverse effects. It could be used in LSC expansion therapy [16]. The report of using AMEED had no adverse effects and the recommendation of using for LSC expansion therapy in this study is in accordance with our investigation. Comparing to the AMEED study, AF study also demonstrated increasing LSC proliferation and accelerating corneal epithelium healing without adverse effects.

**Table 2:** Dry Eye Signs and Symptom Before and After the Treatment With Amniotic Fluid in the Two Study Groups

Time of Assessment	Evaluated Sign or Symptom								
	Corneal staining	TBUT	Pain	Red eye	Dry feeling	BOV	Secondary tearing	Itchiness	Mucus discharge
	n (%)	n (%)	n (%)	n (%)	n (%)	n (%)	n (%)	n (%)	n (%)
	**Group 1**								
Before treatment	Diffuse,12 eyes (100%)	< 5 S,12 eyes (100%)	Severe 12 eyes (100%)	Severe, 12 eyes (100%)	Severe, 12 eyes (100%)	Yes, 12 eyes (100%)	Severe, 12 eyes (100%)	Severe, 12 eyes (100%)	Severe, 12 eyes (100%)
After Treatment with improvement	Localize, 5 eyes (42%)	>5 S, 6 eyes (50%)	Mild, 6 eyes (50%)	None, 6 eyes (50%)	None 6 eyes (50%)	Improve, 6 eyes (50%)	None, 6 eyes (50%)	None, 6 eyes (50%)	None, 6 eyes (50%)
	**Group 2**								
Before treatment	Diffuse, 10 eyes (100%)	< 5 S, 10 eyes (100%)	Severe, 10 eyes (100%)	Severe, 10 eyes (100%)	Severe, 10 eyes (100%)	Yes, 10 eyes (100%)	Severe, 10 eyes (100%)	Severe, 10 eyes (100%)	Severe, 10 eyes (100%)
After treatment with improvement	Localize, 5 eyes (50%)	>5 S, 5 eyes (50%)	Mild, 5 eyes (50%)	None, 5 eyes (50%)	None, 5 eyes (50%)	Improve, 5 eyes (50%)	None, 5 eyes (50%)	None, 5 eyes (50%)	None, 5 eyes (50%)

**Group 1: Used Soft Contact Lens soaked in Amniotic Fluid (FloGraft). **

**Group 2: Used 6 mL of artificial tears mixed with 0.25 mL of Amniotic Fluid (FloGraft). **

**TBUT: Tear break up time; BOV: Blurring of Vision; n: number; %: percentage, S: second. **

We believe this is the first study using AF to treat severe DED. Artificial tears alone cannot provide sufficient treatment for this level of DED [2]. Soft contact lens alone may provide some improvement for some cases; however, it may not be sufficient for some others. The study using cryopreserved amniotic membrane (PROKERA) presented by McDonald et al. demonstrated 88% improvement of ocular surface and reduction of the DEWS score at one week [17]. However, it is very costly, uncomfortable and affect vision during the treatment (Table 1). This case series demonstrated the safety and some added effectiveness in treating severe DED with AF (FloGraft) combined with artificial tears or SCL in short term without the concern of excessive cost, uncomfortableness and poor vision. This study is limited by a small population in a single center with non-significant difference outcomes between the two treatment arms. However, both groups were treated with AF with improvement in symptoms and signs. The strength is no adverse effects in this study for the AF. Future long-term multicenter large population prospective control studies are needed to validate the efficacy. 

## CONCLUSIONS

Amniotic fluid (FloGraft) may be safe to mix with artificial tears or soaked with SCL to treat severe DED based on findings of this study. It improved short term symptoms and signs of severe DED in this case series by about 50% without adverse effects.
